# Antioxidant mitoquinone ameliorates EtOH-LPS induced lung injury by inhibiting mitophagy and NLRP3 inflammasome activation

**DOI:** 10.3389/fimmu.2022.973108

**Published:** 2022-08-18

**Authors:** Wenhua Sang, Sha Chen, Lidan Lin, Nan Wang, Xiaoxia Kong, Jinyan Ye

**Affiliations:** ^1^ School of Basic Medical Sciences, Institute of Hypoxia Research, Cixi Biomedical Institute, Wenzhou Medical University, Wenzhou, China; ^2^ School of Basic Medical Sciences, Zhejiang University, Hangzhou, China; ^3^ Department of Respiratory Medicine and Critical Care Medicine, The First Affiliated Hospital of Wenzhou Medical University, Wenzhou, China

**Keywords:** acute lung injury, lipopolysaccharide, NLRP3 inflammasome, mitophagy, reactive oxygen species

## Abstract

Chronic ethanol abuse is a systemic disorder and a risk factor for acute respiratory distress syndrome (ARDS) and chronic obstructive pulmonary disease (COPD). However, the mechanisms involved are unknown. One explanation is that ethanol produces damaging reactive oxygen species (ROS) and disturbs the balance of mitochondria within the lungs to promote a pro-injury environment. We hypothesized that targeting an antioxidant to the mitochondria would prevent oxidative damage and attenuate EtOH-LPS-induced lung injury. To test this, we investigated the effects of mitochondria-targeted ubiquinone, Mitoquinone (MitoQ) on ethanol-sensitized lung injury induced by LPS. Lung inflammation, ROS, mitochondria function, and mitophagy were assessed. We demonstrated that chronic ethanol feeding sensitized the lung to LPS-induced lung injury with significantly increased reactive oxygen species ROS level and mitochondrial injury as well as lung cellular NLRP3 inflammasome activation. These deleterious effects were attenuated by MitoQ administration in mice. The protective effects of MitoQ are associated with decreased cellular mitophagy and NLRP3 inflammasome activation *in vivo* and *in vitro*. Taken together, our results demonstrated that ethanol aggravated LPS-induced lung injury, and antioxidant MitoQ protects from EtOH-LPS-induced lung injury, probably through reducing mitophagy and protecting mitochondria, followed by NLRP3 inflammasome activation. These results will provide the prevention and treatment of ethanol intake effects with new ideas.

## Introduction

Acute lung injury (ALI) presents a pervasive health burden due to high morbidity. Along with its most severe form, acute respiratory distress syndrome (ARDS), ALI is a disorder of acute inflammation and tissue injury that is characterized by the loss of alveolar-capillary membrane integrity, excessive neutrophil migration, and release of pro-inflammatory and cytotoxic mediators (1–4). The main cause of ALI is direct lung injury, such as inhalation of toxic substances, and ALI is also induced by indirect systemic diseases, including sepsis, bacterial pneumonia, and severe trauma (5).

Chronic alcohol abuse is a systemic disorder and has been widely recognized as a risk factor for developing lung disorders (6). Previously, clinical statistics show that chronic alcohol abuse and smoking are more likely to cause acute lung injury, and that lung injury tends to be more severe when it occurs (7). And reports have also shown that alcohol abuse increases the incidence of ARDS and that chronic ethanol ingestion in mice depletes alveolar epithelial glutathione as well as increases lipopolysaccharide (LPS)-mediated lung edema (8). Therefore, it is important to understand the molecular mechanisms by which ethanol worsens LPS-induced lung injury and to develop therapies that prevent further disease progression.

Ethanol and its metabolites inhibit the immune response of alveolar macrophages (AMs), increase airway leakage, produce damaging reactive oxygen species (ROS), and disrupt the balance of antioxidants/oxidants within the lungs (9). Ethanol promotes oxidative stress by increasing ROS formation as well as decreasing cellular defense mechanisms. Binge drinking results in mitochondria oxidative damage, which induces mitochondrial dysfunction (10, 11). Evidence supports that altered regulation of mitochondrial dynamics might be a common pathogenic pathway of ethanol damage (12, 13). Meanwhile, mitochondrial injury and ROS production activate nuclear transcription factors (e.g., NF-κB) and inflammasome pathways, promote the inflammatory response, and cause cellular damage, leading to organ dysfunction (14–16). The NLRP3 inflammasome is a major intracellular multiprotein complex of the innate immune system. Its components include the NOD-like receptor, NLRP3 protein, adaptor ASC protein, and caspase-1. Upon endogenous and exogenous factor stimuli, components are recruited to form an inflammasome, which processes cytokine precursors (pro-IL-1β and pro-IL-18) into their active and secreted forms (17, 18). Recent studies have shown that chronic ethanol exposure promotes hyper-activation of the NLRP3 inflammasome in CE J774 macrophage cells (19). Activation of the NLRP3 inflammasome by cholesterol crystals in alcohol consumption induces atherosclerotic lesions (20). Meanwhile, mitochondria and ROS production also play important roles in the activation of the NLRP3 inflammation body in ALI (21–23). Inhibition of inflammasome activation improves lung acute injury induced by carrageenan (24). However, whether mitochondria ROS production is related to the NLRP3 inflammasome in ethanol-sensitive ALI is unclear.

Mitophagy leads to selective autophagic degradation of damaged mitochondria and cytolysosome selective packaging of damaged mitochondria. An alcoholic liver disease study showed that mitophagy can remove damaged mitochondria and thus maintain the normal physiological function of cells as well as protect cells (25, 26). However, another study noted that in chronic ethanol treatment of hepatic stellate cells from mice, alcohol activates autophagy, which further aggravates liver pathological changes in the liver fiber (18). Recently, mitochondria-selective autophagy, mitophagy, has been shown to play a key regulatory role in CSE-induced mitochondrial ROS production in chronic obstructive pulmonary disease (COPD) pathogenesis (27, 28). In addition, mitophagy, has emerged as a central player in maintaining mitochondrial homeostasis through elimination of damaged mitochondria, leading to prevention of hyper inflammation triggered by NLRP3 inflammasome activation (29, 30). However, whether the pathology of ethanol-LPS-induced lung injury is involved in mitophagy, and the effect of mitophagy in this process, is unclear.

MitoQ is a widely studied and applied mitochondrial-targeted antioxidant, and it is composed of TPP^+^ and coenzyme Q10 (31). Recent studies indicate that MitoQ accumulates in the mitochondrial matrix and that it is continually reduced by the respiratory chain to its active form and protects mitochondria from oxidative damage (32). *In vitro* and *in vivo*, MitoQ is protective against many oxidative damage-related pathologies, including ischemia-reperfusion injury (33), cardiovascular diseases (34), ethanol-dependent hepatosteatosis (31), and sepsis (34). Meanwhile, another study showed that mitochondria-targeted antioxidant MitoQ ameliorates experimental mouse colitis by suppressing NLRP3 inflammasome-mediated inflammatory cytokines (35). Therefore, it is necessary to better understand the protective mechanisms of MitoQ in mediating mitochondrial ROS formation and the consequent effects on cell signaling.

In this study, the mitochondria-targeted antioxidant MitoQ was added to suppress the mitochondria ROS signaling pathways to evaluate the protective effect of MitoQ on ethanol-LPS-induced acute lung injury in mice. Our data demonstrated that MitoQ inhibited mitochondrial injury and production of ROS as well as maintained the mitochondrial membrane potential. MitoQ further inhibited NLRP3 inflammasome-mediated inflammatory cytokines, which are related to inhibition of mitophagy. These results suggest a protective role of MitoQ on ethanol-LPS-induced acute lung injury. This study provides therapeutic targets for the treatment of ethanol worsening lung injury induced by LPS.

## Material and methods

### Animals and experimental protocol

6-8-wk-old, male C57BL/6 mice (20-25g) were obtained from the Animal Center of the Chinese Academy of Science (Shanghai, China). The mice were randomly divided into five groups (Control group, EtOH group, LPS group, EtOH+LPS group, MitoQ+EtOH+LPS group). Each group contained 25 mice. They were maintained at 22°C with a 12h light/dark cycle and had free access to a normal chow diet and tap water for 3 days. For EtOH group, EtOH+LPS group, and MitoQ+EtOH+LPS group, ethanol was added in the drinking water gradually increased from 5% (v/v) to 15% (v/v) in first week, and remained at 20% for subsequent 4 weeks. LPS inhaled at a dose of 5 mg/kg in saline *via* nasal cavity for 6 hours was conducted on the last day in the morning. Ethanol-fed mice were given saline by nasal inhalation. MitoQ was purchased from MedChemExpress (HY-100116A). MitoQ(10mg/ml) was dissolved in an ethanol-water mixture with a ratio of 1:1 as the storage solution. For MitoQ+EtOH+LPS group, MitoQ (10mg/kg) was given by intraperitoneal injection twice a week since beginning of ethanol. Control-fed mice were maintained on standard chow and water. With these general approaches, the blood alcohol levels of animals following chronic ethanol consumption has been reported to be 0.08% (36, 37).

### Establishment of acute lung injury (ALI) mice model

At the end of the experiment, the mice were anesthetized by intraperitoneal injection of 100mg/kg pentobarbital sodium. Six mice were randomly selected from each group, then lung samples were harvested aseptically for subsequent experiments. Lung samples were divided into three parts, one of which was stored at -80°C for protein isolation and tissue homogenate. The upper left lung lobe from each animal was immediately fixed in 4% paraformaldehyde overnight. Lungs were gradually dehydrated, embedded in paraffin, cut into 4μm sections, and stained with hematoxylin and eosin (H&E). The other part lungs used OCT embedding immediately frozen by liquid nitrogen, and cut into 6μm sections for DHE dyeing.

### Measurement of lung W/D weight ratio

The magnitude of pulmonary edema was evaluated by the W/D ratio of lung tissues. Six mice were randomly selected from each group, the left lung lobe was removed, rinsed with saline, and weighed to obtain the wet weight. The lung was then dried at 60°C for 72h and weighed to obtain the dry weight. The W/D ratio was then calculated.

### Enzyme-linked immunosorbent assay (ELISA)

Six mice were randomly selected from each group for collected bronchoalveolar lavage fluid (BALF). BALF was collected from the lung by intratracheal injection of 0.8 ml of ice-cold sterile 0.9% saline for three times followed by gentle aspiration. The recovered BALF fractions were pooled and centrifuged (1000×g, 10min, 4°C) using a cooling centrifuge to collect the cell pellet for total cell count determination. The cell-free supernatant of BALF was stored at −80°C for assessment of TNF-α, IL-6, IL-1β by ELISA, and total protein by BCA.

### Cell culture and drug treatment

Mouse macrophage cell line Raw264.7 and human carcinoma A549 cells was obtained from American Type Culture Collection (ATCC). Cells were cultured in DMEM supplemented with 10% fetal bovine serum and 1% penicillin/streptomycin and were maintained at 37°C and 5% CO2. Human Umbilical Vein Endothelial Cells (HUVEC) was obtained from Wuhan Punosai Life Technology. HUVEC were cultured in ECM supplemented with 10% fetal bovine serum, 1% endothelial growth factor and 1% penicillin/streptomycin. Cells were used from 10 to 15 passages and cultivated in 6-well culture well plates at a starting seeding density of 5×10^5^ cells. Overnight, cells were treated for experiment, 25mM ethanol for 48h (ethanol medium was changed at each 24h), then followed by LPS (1μg/mL) for 6h, and the control group was given the same dose PBS. The 25mM *in vitro* ethanol concentration approximates a 0.1g/dl blood alcohol level (37) which is accordant with a dose of moderate drink *in vivo*. For MitoQ+EtOH+LPS group, Raw264.7 cells were pretreated with MitoQ 100ng/mL (dissolved in PBS) at the beginning of ethanol.

### ELISA for IL-1β cytokines in cell culture medium

Raw 264.7 cells (2 × 10^5^ per well) were seeded in 24-well plates containing DMEM supplemented with 10% FBS for 1 day to become nearly confluent. On the second day, the cells were pretreated with or without 25mM ethanol for 48h before treatment with LPS at 37°C for 6h. The cultured media were collected and Immunoreactive IL-1β in cultured media were measured by ELISA development kits according to the manufacturer’s instruction (Dakewe biotech).

### Measurement of mitochondrial membrane potential

In this experiment, mitochondrial membrane potential (ΔΨm) of Raw264.7 cells were determined by Rhodamin 123 assay as described previously. Briefly, after treatment, the cells were loaded with 10μM Rho123 for 30 min at 37°C. Then wash the plates with PBS for three times. The fluorescence of Rho123 was excited at 488nm and the emission was collected at 526 nm by using a fluorescence plate reader. ΔΨm was measured in isolated mitochondria (mouse lung) by using the cationic dye 5, 5’, 6, 6’-tetrachloro-1, 1’, 3, 3’-tetraethylbenzimidazolylcarbocyanineiodide (JC-1). Isolated intact mitochondria were incubated with 10μmol/L JC-1 for 30 minutes at 37°C. JC-1 monomers emit green fluorescence, but when they enter live mitochondria, they form J-aggregates, which emit red fluorescence. We measured the fluorescence by using the fluorescence plate reader. Mitochondrial membrane potential (ΔΨm) was also measured by microscopy with JC-1 staining. After drug treatment, JC-1 was added to cells and further incubated for 30min at 37°C. The lost ΔΨm was detected by using an inverted fluorescence microscope. In normal circumstances, mitochondrial appears red fluorescence, and green fluorescent means membrane potential decline.

### Western blotting

Proteins were extracted from the lung tissue samples, Raw264.7 cells, A549 cells and HUVEC cells using Radio Immunoprecipitation Assay (RIPA) buffer (Beyotime, P0013B) with PMSF lysis buffer. The protein from each condition was spectrophotometrically quantified (Micro BCA Protein Reagent Kit, Beyotime), and an equal amount of protein (30-90μg) was loaded onto 10%-15% SDS-PAGE gels. After electrophoresis, proteins were transferred to PVDF membranes (Immobilon, IPVH00010). Membranes were blocked in 5% non-fat milk in Tris-buffered saline (TBS) for 1.5 h and probed with antibodies. The membranes were incubated with Tom20 (Santa, 1:500, sc17764), NLRP3 (CST,1:1000, 15101), GAPDH (bioworld, 1:50000, AP0063), Anti-Bcl-2 (Abcam, 1:2000, ab196495), Anti-Bax (Abcam,1:10000, ab32503), Cleaved-Caspase-3 (CST, 1:1000, 9664), Drp-1 (Abcam, 1:1000, ab184247), Nrf2 (CST,1:1000, 12721), Caspase-1 (CST, 1:1000, 2225), IL-1β (CST, 1:1000, 12426), LC3A/B (Abcam, 1:1000, ab128025), SQSTM/p62 (Abcam, 1:1000, ab56416), PINK1 (Santa Cruz, 1:1000, sc-33796), Parkin (Abcam, 1:1000, ab15954), Mfn1(CST, 1:1000, 14739). After three washes with TBST, the membranes were incubated with horseradish peroxidase-conjugated anti-rabbit or anti-mouse secondary antibodies for 1h at room temperature. The relative intensities of bands were analyzed by using image analysis software program (Image J 1.44p, Wayne Rasband, National Institutes of Health, USA).

### Determination of free intracellular [Ca2+]

The free intracellular [Ca2+] in Raw264.7 cells were measured using the fluorescence Ca2+ indicator, fura-2/AM, as previously reported (38). First, Raw264.7 cells were seeded in 24-well plates (3× 10^5^/per well). After pretreated with or without 25 mM ethanol for 48 h before treatment with LPS at 37 °C for 6 h. Fura-2 AM (5 μg/mL) was then added to the cells. After 45 min cells were rinsed twice with 2% BSA/PBS and incubated another 10 min at 37°C before fluorescence intensities at 340 nm (F340) or 380 nm (F380) were detected simultaneously by microplate reader. The [Ca2+]i was calculated as follows: [Ca2+]i=Kdβ (R− Rmin)/(Rmax− R), where β is the ratio of F380max (the fluorescence emission at 380 nm at a zero level of free Ca2+) to F380min (the fluorescence intensity at a saturating level of free Ca2+) and R represents the fluorescence ratio of F340 and F380. The maximal fluorescence ratio (Rmax) was determined after adding Triton X-100 (0.1%) and the minimal fluorescence ratio (Rmin) was determined by adding EGTA (5 mM; Sigma). After determining [Ca2+]i, cells were lysed in 20 μL of 0.1 M NaOH and total cellular protein concentrations were measured using the BCA Assay method. [Ca2+]i was normalized to total cellular protein and presented as nmol/mg protein.

### Detection of cellular ATP levels

Cellular ATP levels were measured using a firefly luciferasebased ATP assay kit (Beyotime, China) according to the manufacturer’s instructions. Cells were seeded in 24-well plates (3× 10^5^/per well), after treatment cells were schizolysised and centrifuged at 12,000 ×g for 5 min. 100 μL of each supernatant was mixed with 100 μL ATP detection working dilution. Luminance (RLU) was measured by microplate reader. Standard curves were also generated and the protein concentration of each treatment group was determined using the Bradford Protein assay. Total ATP levels were expressed as nmol/mg protein.

### Immunofluorescence microscopy analysis

For lung tissue, after deparaffinization, serum blocking, and antigen retrieval, sections underwent immunofluorescence staining with primary antibodies against F4/80 (Abcam, 1:200), NLRP3 (CST, 1:200), LC3 (Santa, 1:200) at 4°C overnights. Alexa Fluor 488- or 594- conjugated secondary antibodies (Molecular Probes, Carlsbad, CA) were added to sections for 1h at room temperature. Treated with 4’, 6-diamidino-2-phenylindole (DAPI) for 5 min was used for nuclear counterstaining. Sections were mounted with a coverslip, sealed with nail polish, and stored in the dark at 4°C.

Raw246.7 cells were seeded in 6-well plates containing coverslips overnight before indicated treatments. After the treatment, cells were washed with PBS and fixed with 4% PFA in PBS for 20 min at room temperature, and then permeabilized with 0.5% Triton X-100 in PBS for 15 min and blocked with 5% BSA in PBS for 45 min at room temperature. After blocking, cells were incubated with rabbit monoclonal antibody to NLRP3 (CST, 1:200) overnight at 4°C. After further washed three times with PBS, cells were incubated with TRITC-conjugated goat anti-rabbit IgG (1:200). The nucleus was stained with DAPI. All images were captured on Nikon ECLIPSE Ti microscope (Nikon, Tokyo, Japan).

### Measurement of mitochondrial superoxide

The level of mitochondrial superoxide was detected using MitoSOX red mitochondrial superoxide indicator. MitoSOX Red, is a redox-sensitive fluorescent probe that is selectively targeted to the mitochondria’s ROS. Raw264.7 cells were incubated with 5 μmol/L MitoSOX for 30 minutes at 37°C and 5% CO_2_ after LPS treatment for 6 h and 25mM EtOH treatment for 48 h. Cells were then detached by gentle scraping with a cell scraper and analyzed by fluorescence microplate. The fluorescence of MitoSOX was excited at 356nm and the emission was collected at 580 nm.

### Statistical analysis

Results are presented as means ± SE of each group. A Student’s t-test was used to determine statistical significance between two groups. Statistical analysis was performed by a one-way ANOVA test and values of P < 0.05 were considered statistically significant.

## Results

### MitoQ decreases ethanol-LPS-induced pathological changes in acute lung injury

**Figure 1:**
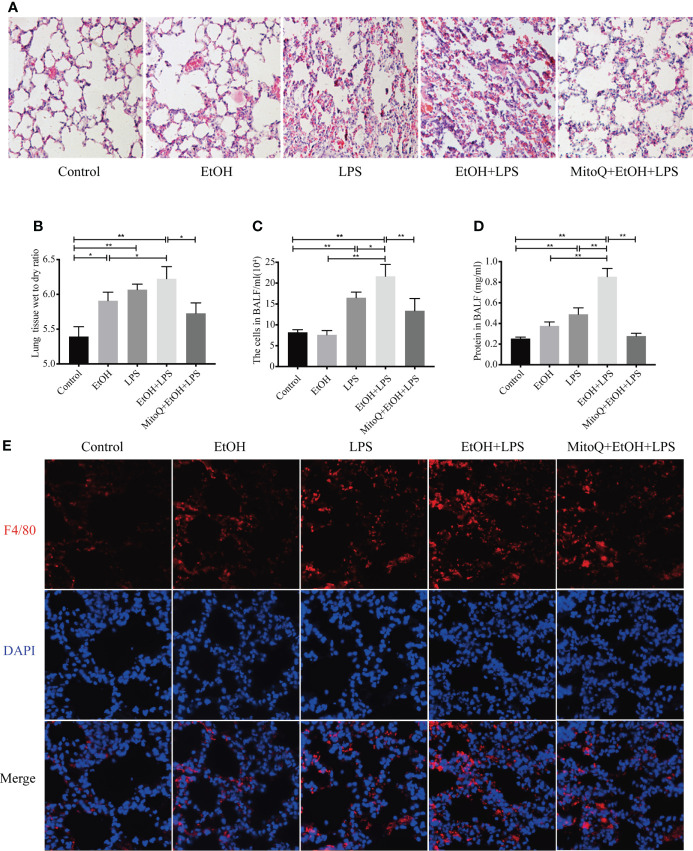
MitoQ alleviated lung injury induced by ethanol and LPS. C57BL/6 mice were pretreated with or without gradient alcohol feeding 5 weeks before LPS (5 mg/kg) inhaled for 6 h. Intraperitoneal injection MitoQ (10 mg/kg) twice a week at beginning of ethanol. **(A)** Representative histological section of the lungs was stained with hematoxylin and eosin (H&E staining magnification 200×). **(B)** The left lung tissues were weighed before and after being dried at 60 °C for 72 h. The W/D ratio was then calculated. **(C)** The BALF was collected to measure the number of total cells. **(D)** The supernatant of BALF was collected and used BCA to measure protein content. **(E)** Immunostaining of F4/80 (red) was analyzed in lung tissues (magnification 400×). All data were presented as mean ± SEM (n=6 in each group), *P < 0.05, **P < 0.01.

To investigate the effect of ethanol on lung injury induced by LPS, the pathological changes of lung tissue in each group were evaluated by microscopy. As shown in **Figure 1**, ethanol or LPS alone increased inflammatory cell infiltrates in the lung interstitium and in LPS group alveolar spaces increased alveolar wall thickening. A hemorrhage was observed in hematoxylin and eosin (H&E)-stained sections. Compared with the single ethanol or LPS group, ethanol exposure significantly thickened the alveolar interval and increased the inflammatory cell infiltrates induced by LPS in the lung tissue. MitoQ, a mitochondria-target antioxidant, was administered at the beginning of ethanol treatment. Interestingly, MitoQ is markedly protected against lung injury induced by ethanol and LPS in mice (**Figure 1A**). The lung W/D weight ratio is a commonly used parameter for assessing experimental lung edema. Ethanol or LPS alone increased the lung W/D ratio. Lung edema is significantly worse in Ethanol/LPS than Ethanol group. Treatment with MitoQ obviously decreased the lung W/D ratio induced by ethanol and LPS (**Figure 1B**). To further identify the effect of ethanol on the pro-inflammatory factors induced by LPS, the number of total cells that infiltrated the lungs was determined in BAL fluid. Chronic ethanol increased LPS-induced cell seeping and the numbers of total cells in BALF were increased compared to the control group. Ethanol exposure alone did not significantly affect the cell and protein levels in the lung in the absence of LPS, but remarkably enhanced the effect of LPS. Furthermore, MitoQ obviously decreased the numbers of total cells and protein levels in BALF by ethanol and LPS (**Figures 1C, D**). Immunofluorescence staining in F4/80 revealed that a single ethanol exposure or LPS increased the number of macrophages, and there was no significant difference between the ethanol and LPS groups, but ethanol significantly increased the numbers of macrophages in lung samples induced by LPS. Interestingly, MitoQ remarkably decreased the numbers of macrophages (**Figure 1E**). These results indicated that ethanol worsens lung injury induced by LPS, which was obviously alleviated by MitoQ.

### MitoQ administration reduces generation of inflammatory cytokines of acute lung injury induced by ethanol-LPS in BALF

**Figure 2:**
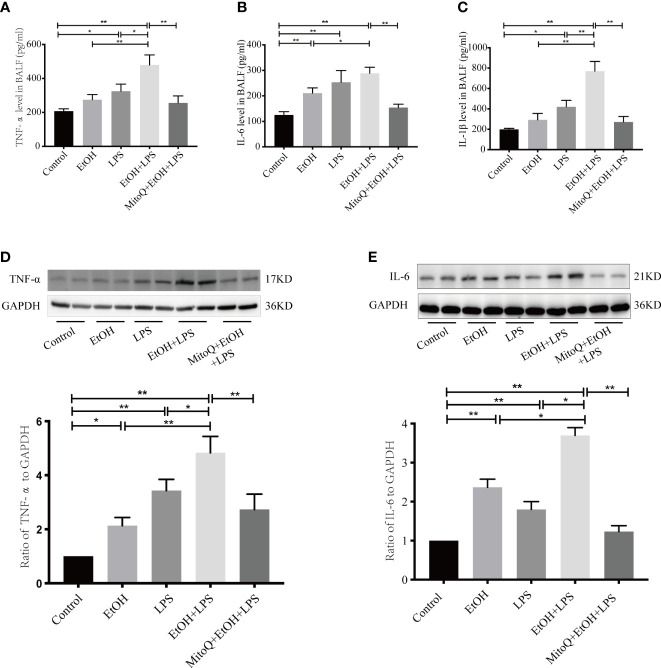
MitoQ decreased ethanol-LPS-induced pro-inflammatory factor production. The BALF were pooled and centrifuged (1000×g, 10 min, 4°C). The cell-free supernatant of BALF was assessed for TNF-α **(A)**, IL-6 **(B)**, and IL-1β **(C)** by ELISA. The pro-inflammatory factor in lung tissue protein TNF-α **(D)**, and IL-6 **(E)** was measured by western blotting. All data were presented as mean ± SEM (n=6 in each group), *P < 0.05, **P < 0.01.

To further evaluate the effect of ethanol and LPS on inflammation in lung tissue, inflammatory mediators in BALF were measured by ELISA. As shown in **Figure 2**, chronic ethanol ingestion alone caused a small but not statistically significant increase in the levels of TNF-α and IL-1β, but the IL-6 levels were obviously increased in the ethanol group. Compared to control animals, LPS significantly increased the levels of TNF-α, IL-6, and IL-1β in the BALF. Compared to the ethanol or LPS group, chronic ethanol enhanced the levels of TNF-α (**Figure 2A**), IL-6 (**Figure 2B**), and IL-1β (**Figure 2C**) in BALF induced by LPS. MitoQ significantly decreased the levels of TNF-α, IL-6, and IL-1β in BALF. Subsequently, protein expression of key inflammatory cytokines (TNF-α, IL-6) in lung tissue was determined by western blotting. Similar results were observed in the protein expressed from lung tissue. Ethanol or LPS alone increased expression of TNF-α and IL-6. Chronic ethanol feeding enhanced the protein expression of TNF-α and IL-6 (**Figures 2D, E**) induced by LPS in lung tissue, which was significantly decreased by MitoQ. These results indicated that MitoQ reduced the level of inflammatory cytokines released by inflammatory cells.

### MitoQ decreases NLRP3 inflammasome activation induced by ethanol and LPS in lung tissue

**Figure 3:**
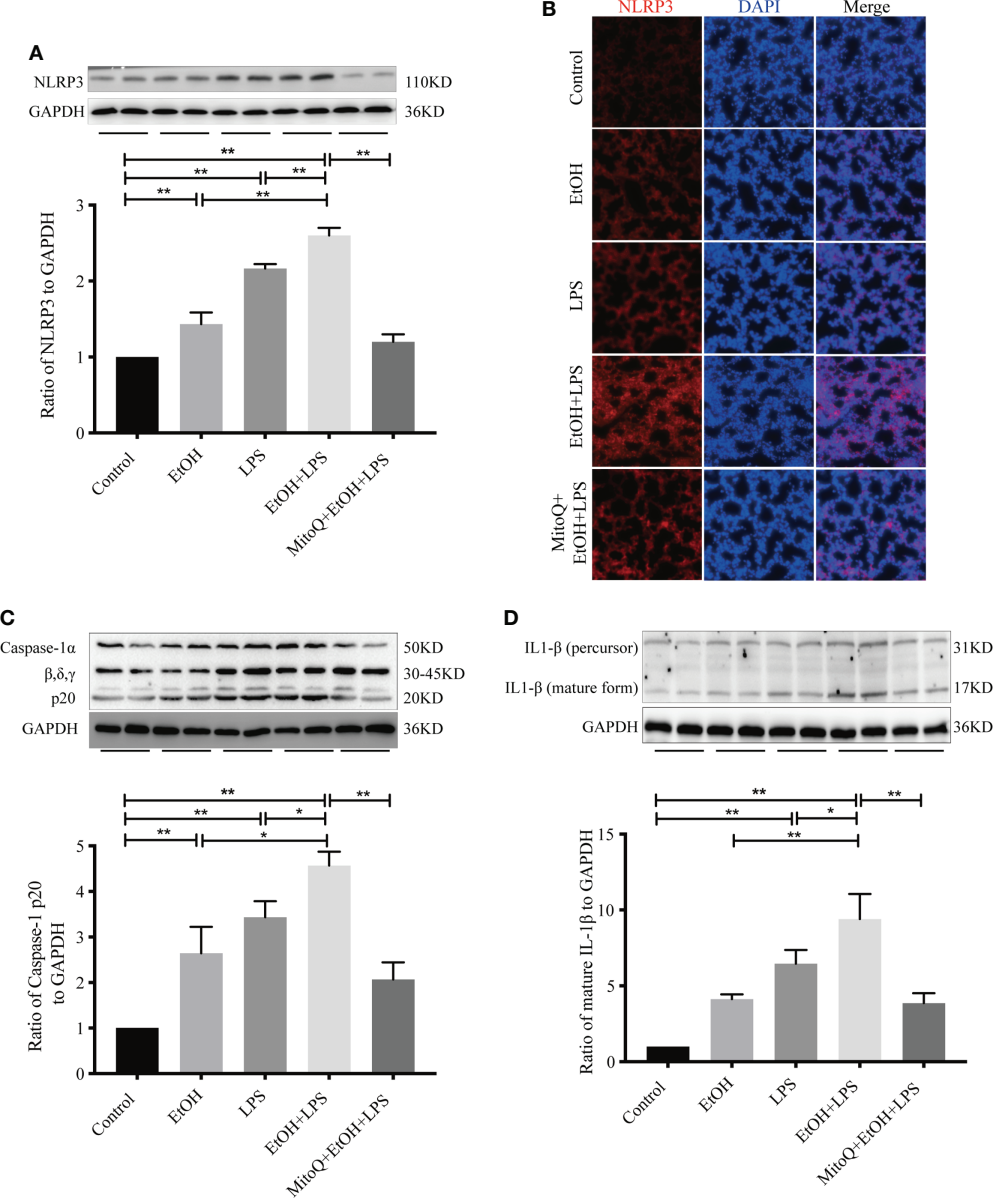
MitoQ decreased ethanol-LPS-induced NLRP3 inflammasome activation. **(A–D)** Effect of NLRP3/IL-1β Signaling pathways in the model of C57BL/6 mice. NLRP3**(A)**, Caspase-1**(C)**, and IL-1β**(D)** in lung tissue total proteins were detected by western blotting. **(B)** Protein NLRP3 (red) activation on the lung tissue section was analyzed by immunofluorescence (magnification 400×). All data were presented as mean ± SEM (n=6 in each group), *P < 0.05, **P < 0.01.

The NLRP3 inflammasome is one of the best understood members of the inflammasome family, and it is expressed at high levels in macrophages (39). The NLRP3 inflammasome activates caspase-1, leading to maturation of IL-1β and IL-18. IL-1β plays a critical role in the acute phase response and sepsis. We next tested expression of NLRP3 in lung tissue after treatment with ethanol and LPS and (or) MitoQ. As shown in **Figure 3A**, compared with the control group, ethanol or LPS alone significantly increased the protein levels of NLRP3 in lung tissue. Chronic ethanol ingestion remarkably increased the expression of NLRP3 induced by LPS. Importantly, treatment with MitoQ significantly reduced the protein levels of NLRP3 in lung tissue in the ethanol and LPS groups. Subsequently, the NLRP3 protein was analyzed using immunofluorescence staining, and we found results that were consistent with those seen in lung tissue (**Figure 3B**). Then, expression of Caspase-1α and IL-1β was detected by western blotting. Ethanol or LPS alone caused a small increase in protein expression of active caspase-1α and IL-1β, and chronic ethanol exposure enhanced the protein levels of active caspase-1α and IL-1β induced by LPS. Interestingly, treatment with MitoQ markedly decreased NLRP3 inflammasome activity and also decreased caspase-1α and IL-1β activities, as shown in **Figures 3C, D**. These data show that MitoQ administration reduces ethanol-LPS-induced activation of the NLRP3 inflammasome.

### MitoQ protects ethanol-LPS-increased ROS production and mitochondria injury

**Figure 4:**
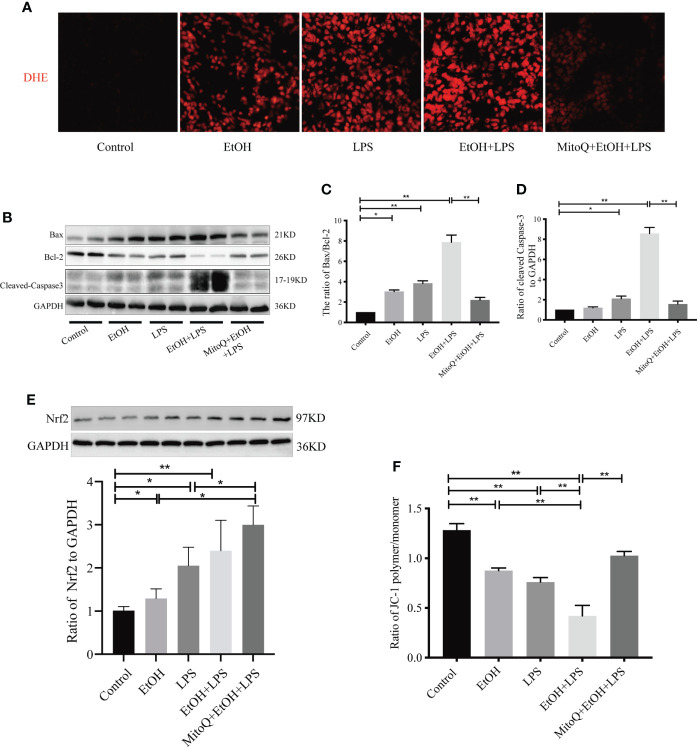
MitoQ decreased ethanol-LPS-induced ROS production and mitochondria injury. **(A)** DHE staining (red) was performed to assess ROS levels. **(B)** Protein expression of Bax, Bcl-2, and Cleaved Caspase-3 in lung tissue was detected by western blotting. **(C)** Relative expression of protein bax/bcl-2 was normalized against GAPDH. **(D)** Relative expression of protein Cleaved Caspase-3 was normalized against GAPDH. **(E)** Relative expression of protein Nrf2 was normalized against GAPDH. **(F)** Mitochondrial membrane potential (ΔΨm) was evaluated by the OD value of fluorescence microplate and then calculated the ratio of polymer/monomer. All data were presented as mean ± SEM (n=6 in each group), *P < 0.05, **P < 0.01.

The mitochondrion is one of the important organelles, and maintaining intracellular capacity for cell physiological function is necessary. To determine whether oxidative stress is involved in the ethanol-worsened lung injury induced by LPS, as shown in **Figure 4A**, we evaluated ROS production in frozen sections of lung tissues by DHE staining. Both ethanol and LPS increased ROS production. Chronic ethanol feeding significantly elevated ROS production by LPS. Interestingly, there was a significant decrease in the MitoQ+EtOH+LPS group. Bax/Bcl-2 activation remains a central question in mitochondria-dependent apoptotic signaling. To assess mitochondrial injury after ethanol or/and LPS and the protective role of MitoQ, the change in Bax and Bcl-2 protein levels was detected by western blotting. As shown in **Figures 4B–D**, ethanol or LPS alone increased the expression ratio of Bax/Bcl-2 and Cleaved Caspase-3 protein. Chronic ethanol significantly aggravated the changes induced by LPS. As expected, MitoQ dramatically ameliorated the ratio of Bax/Bcl-2 and expression of Cleaved Caspase-3 protein. The expression of Nrf2 protein **(** **Figure 4E** **)** showed endogenous antioxidant pathways being activated *in vivo* after feeding ethanol and LPS inhalation. MitoQ further increased the expression of Nrf2 protein induced by EtOH+LPS. Next, the mitochondrial membrane potential (ΔΨm) in mitochondria isolated from lung tissue samples was measured using JC-1 staining and calculated by the ratio of polymer/monomer. As shown in **Figure 4F**, ethanol and LPS alone markedly decreased the levels of ΔΨm. Chronic ethanol significantly aggravated the decrease in the mitochondrial membrane potential induced by LPS. Interestingly, treatment with MitoQ significantly augmented ΔΨm. These results showed that the protective role of MitoQ could be related to amelioration of oxidative stress and maintenance of mitochondrial balance.

### MitoQ decreases ethanol-LPS-induced PINK/Parkin-dependent mitophagy signaling pathway activation in lung tissue

**Figure 5:**
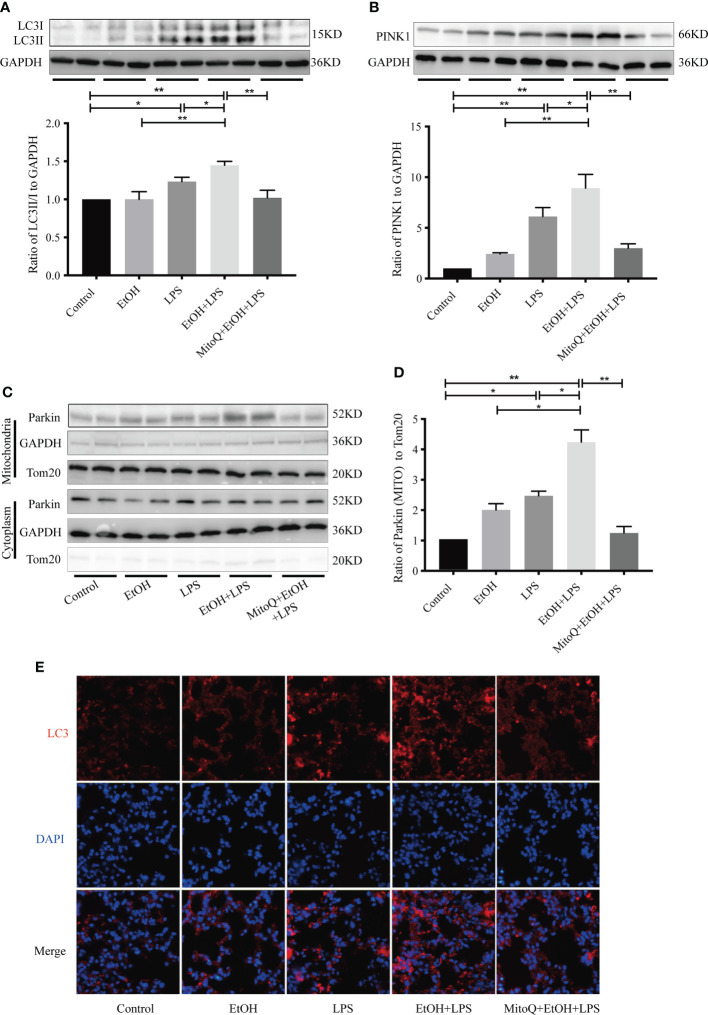
MitoQ inhibited ethanol-LPS-induced mitophagy activation. **(A)** Representative western blot images indicated LC3 protein expression. **(B)** Representative western blot images indicated PINK1 protein expression. **(C)** Expression of Parkin protein in cytoplasm and mitochondria were detected by western blotting. **(D)** Relative expression of the protein was normalized against GAPDH. **(E)** Representative immunohistochemistry images of LC3. All images are shown at identical magnification (×400). All data were presented as mean ± SEM (n=6 in each group), *P < 0.05, **P < 0.01.

Mitochondria are known as the ‘power factories’ of the cell and supply the energy required for cell life. Mitophagy represents selective engulfment of damaged mitochondria by autophagosomes. To determine mitophagy activation in this model, expression of the autophagy-specific marker LC3 was measured in lung tissue. As shown in **Figure 5A**, there was a slight rise in the LC3-II level of the ethanol group, and LPS significantly increased protein expression of LC3-II. However, chronic ethanol exposure markedly increased LPS-induced LC3 expression, which was obviously reduced after the MitoQ injection. Similar results are shown for immunofluorescence staining (**Figure 5E**). To further confirm that chronic ethanol increases autophagy, which is related to the mitochondrial injury induced by LPS, the classic mitophagy signal pathway PINK/Parkin was detected by western blotting (**Figures 5B–D**). Ethanol alone induced a small increase in protein expression of PINK1 and Parkin, LPS alone significantly increased protein expression of PINK1 and Parkin. However, chronic ethanol markedly stimulated an increase in the PINK1 protein level and more Parkin was located in mitochondria, both of which were reversed by MitoQ. These results show that MitoQ maintains the mitochondria balance, which could be related to inhibition of PINK/Parkin-dependent mitophagy.

### MitoQ inhibits ethanol-LPS-induced NLRP3 inflammasome activation in Raw 264.7 cells

**Figure 6:**
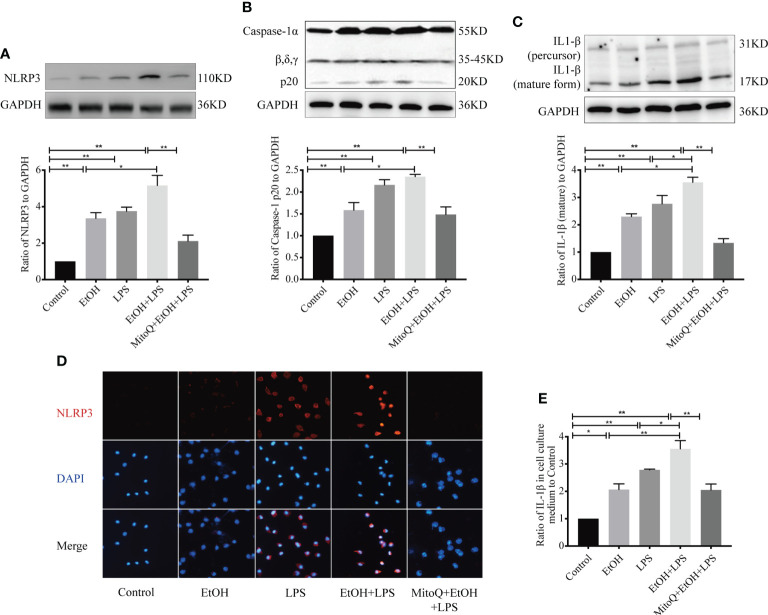
MitoQ decreased ethanol-LPS-induced injury in Raw264.7 cells by reducing NLRP3 inflammasome activation. Protein expression of NLRP3 **(A)**, Caspase-1 **(B)**, and IL-1β **(C)** in the Raw264.7 cell was shown by western blotting. **(D)** Protein NLRP3 (red 400×) inflammasome activation was analyzed by immunofluorescence. **(E)** Protein IL-1β in cell culture medium was detected by ELISA. All data were presented as mean ± SEM (n=5 in each group), *P < 0.05, **P < 0.01.

*In vivo* experiments demonstrated that alcohol intake significantly increased lung macrophages, but MitoQ reversed this phenomenon when suffered EtOH+LPS induced lung damage. It was hypothesized that macrophages play an improtent role in EtOH+LPS induced lung injury. Therefore, macrophages were selected to investigate the mechanism by which MitoQ ameliorates EtOH+LPS-induced lung injury. To further evaluate whether mitochondria ROS play a vital role in regulating NLRP3 inflammasome activation, a chronic ethanol-increased LPS injury model of Raw264.7 cells was used. In agreement with previous experiments, the *in vivo* western blotting results showed that ethanol and LPS increased expression of the proteins NLRP3 (**Figure 6A**), activated caspase-1 (**Figure 6B**), and mature IL-1β (**Figure 6C**) in Raw264.7 cells. Treatment with MitoQ resulted in loss of the NLRP3 protein, activation of caspase-1, and reduced pro IL-1β to the mature form. Subsequently, the expression of the NLRP3 protein was analyzed using immunofluorescence staining, and we found that its expression was consistent with that observed by western blotting (**Figure 6D**). In addition, active IL-1β in the supernatant of the Raw264.7 macrophage cell line was detected by ELISA. We found that IL-1β secretion significantly increased in ethanol or LPS-treated cells and that chronic ethanol exposure markedly increased LPS-induced IL-1β secretion. However, treatment with MitoQ significantly reduced secretion of IL-1β in Raw264.7 cells (**Figure 6E**). These observations are consistent with previous *in vivo* studies and further confirmed that protective role of MitoQ is related to inhibition of NLRP3 inflammasome activation.

### MitoQ protects against the mitochondria injury in duced by ethanol-LPS in Raw264.7 cells

**Figure 7:**
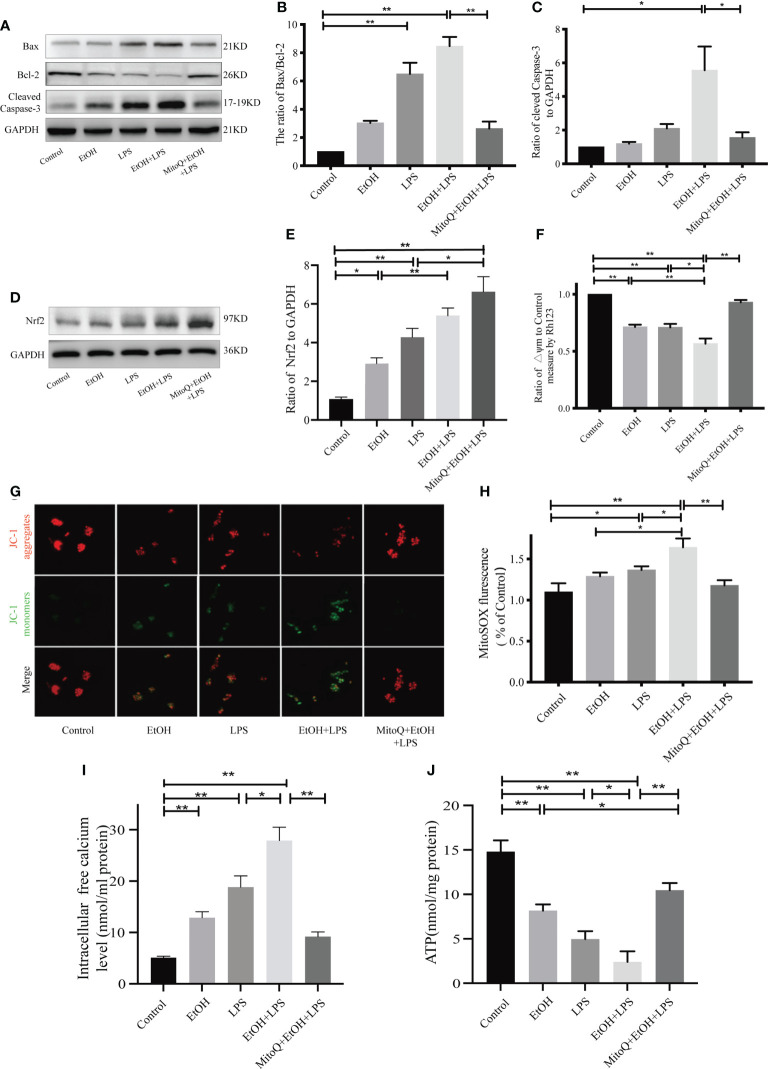
MitoQ protected mitochondria injury in Raw264.7 cells induced by ethanol and LPS. **(A–C)** Protein levels of Bax, Bcl-2, and Cleaved Caspase-3 were detected by western blotting. **(D, E)** Protein levels of Nrf2 were detected by western blotting. **(F)** Mitochondrial membrane potential (ΔΨm) was evaluated by Rho123 fluorescence analysis. **(G)** Mitochondrial membrane potential in Raw264.7 cells were analyzed by JC-1 staining (400×). Red staining indicates polarized mitochondria in JC-1 staining. Green staining indicates depolarized mitochondria in JC-1 staining. **(H)** Mitochondrial ROS was analyzed by fluorescence microplate. **(I)** Intracellular free calcium level was measured using Fura-2/AM in cultures by microplate reader. **(J)** Cellular ATP concentrations were detected in Raw264.7 cells by microplate reader. All data were presented as mean ± SEM (n=5 in each group), *P < 0.05, **P < 0.01.

To confirm that the protective role of MitoQ in lung injury in mitochondria, we analyzed the ratio of Bax/Bcl-2 and protein level of Cleaved Caspase-3 by western blotting (**Figures 7A–C**) in Raw264.7 cells exposed to chronic ethanol-increased LPS injury. Our results showed that ethanol or LPS significantly increased the ratio of Bax/Bcl-2 and protein expression of Cleaved Caspase-3, but chronic ethanol exacerbated LPS-induced increases in the ratio of Bax/Bcl-2 as well as protein expression of Cleaved Caspase-3, which were dramatically decreased by MitoQ in Raw264.7 cells. Similarly, the effect of MitoQ addition on endogenous antioxidant *in vitro* also evaluated. As shown in **Figures 7D, E**, compared to simply EtOH or LPS group, the protein level of Nrf2 in Raw264.7 cells were increased after expose to EtOH+LPS. Treatment with mitoQ further promoted the increased protein level of Nrf2 induced by EtOH+LPS. These observations suggest that MitoQ protects the mitochondrial function induced by ethanol and LPS in Raw264.7 cells.

To further investigate the effects of MitoQ on mitochondrial function, indicators of mitochondrial activity such as membrane potential (Δψm), levels of cellular ATP and intracellular calcium were determined. The mitochondrial membrane potential of Raw264.7 cells was detected by Rh123 staining (**Figure 7F**). Our results showed that both ethanol and LPS decreased ΔΨm. Chronic ethanol significantly aggravated LPS-induced decreases in ΔΨm, which were markedly reversed by MitoQ. Subsequently, ΔΨm was also detected by JC-1 fluorescence, and the same result was found with Rh123 staining. Treatment with MitoQ inhibited the JC-1 fluorescent color change from red to green (**Figure 7G**), which suggested that MitoQ inhibited the decrease of ΔΨm. As shown in **Figure 7J**, ethanol or LPS significantly decreased ATP production, but chronic ethanol exacerbated LPS-induced ATP production decrease, which was dramatically increased by MitoQ. Furthermore, both EtOH, LPS increased intracellular free calcium levels **(** **Figure 7I** **)**, EtOH+LPS further exacerbated calcium leakage from mitochondria to cytoplasm. However, after use of MitoQ significantly reduced the increase of free calcium in cytoplasm due to EtOH+LPS treatment. Consequently, MitoQ attenuated ETOH+ LPS-induced mitochondrial damage in RAW264.7 cells. Subsequently, Mitochondrial ROS was analyzed by fluorescence microplate. Results of Mitochondrial ROS showed that there was a slight rise in mitochondrial ROS of ethanol group, LPS alone significantly elevated ROS production in mitochondria. Ethanol exposure markedly increased LPS-induced mitochondrial ROS level, which were significantly decreased by MitoQ, as shown in **Figure 7H**.

The essential functions of mitochondria have been attributed to their unique dynamic nature: the ability to undergo continuous cycles of fusion and fission that result in changes in mitochondrial morphology. In recent research mitofusins (Mfn1 and Mfn2), and dynamin-related protein 1 (Drp1) were found to be key mediators of mitochondrial fusion and fission (40). In order to determine whether MitoQ can also protect the mitochondrial function of endothelial cells and epithelial cells, we treated A549 cells and HUVEC cells with EtOH+LPS, and detected Drp1 and Mfn1 protein levels by WB **(** **Supplemental Data A, B** **)**. When treated with LPS, the expression of mitochondrial mitotic protein Drp1 was increased and the expression of fusion protein Mfn1 was decreased, which was more significant in the EtOH+LPS group. This phenomenon was observed in both HUVEC cells and A549 cells, suggesting that EtOH increased lPS-induced mitochondrial damage. However, after the application of MitoQ, Drp1 expression was recovered in both HUVEC cells and A549 cells, and the expression of Mfn1 was no different from that of the normal group. This suggested that MitoQ protected the mitochondrial functions of endothelial cells and epithelial cells treated with EtOH+LPS.

### MitoQ decreases ethanol-LPS-induced PINK/Parkin-dependent mitophagy signal pathway activation in Raw264.7 cell

**Figure 8:**
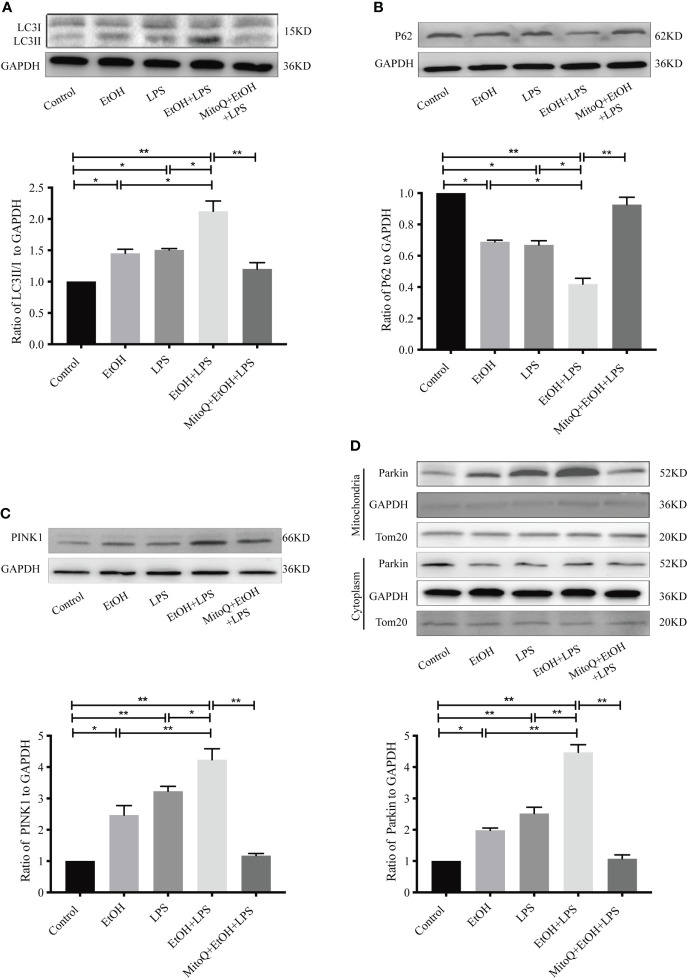
MitoQ inhibited ethanol-LPS-induced mitophagy activation in Raw264.7 cells. **(A)** Expression of LC3 protein was detected by western blotting. **(B)** Representative western blot images indicated P62 protein expression. **(C)** Representative western blot images indicated PINK1 protein expression. **(D)** Expression of Parkin protein in cytoplasm and mitochondria was detected by western blotting. All data were presented as mean ± SEM (n=5 in each group), *P < 0.05, **P < 0.01.

To further demonstrate mitophagy activation *in vivo*, mitophagy-related proteins were detected by western blotting. As shown in **Figures 8A–D**, compared with the control group, Ethanol or LPS alone significantly increased protein expression of LC3-II, PINK1, mitochondrial Parkin and decreased protein expression of p62. However, chronic ethanol intake markedly aggravated LPS-induced protein expression of LC3, PINK1, Parkin, and p62, which were obviously reversed by MitoQ treatment (**Figure 8**). These results suggest that the protective effect of MitoQ on the mitochondrial function induced by ethanol and LPS could be related to inhibition of mitophagy.

### Activation of mitophagy aggravates ethanol-LPS-induced injury in Raw264.7 cells

**Figure 9:**
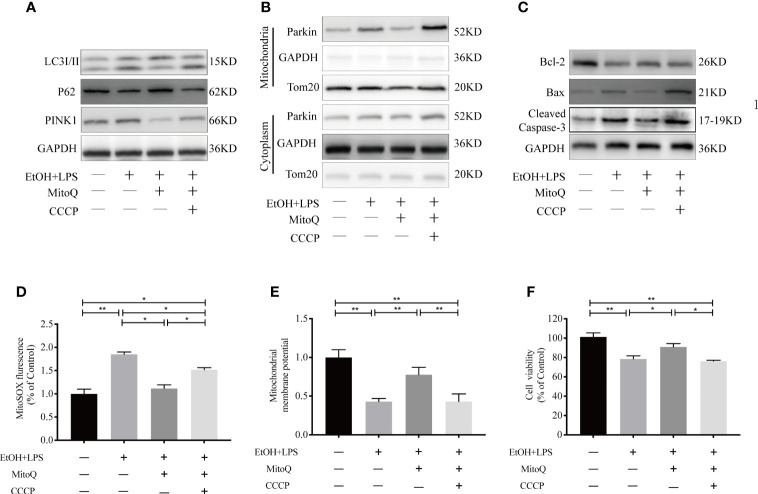
Mitophagy increased ethanol-LPS-induced injury in Raw264.7 cells. Cell were treated with EtOH (25 mM) for 48 h and LPS (1 mg/mL) for 6 h, before the addition of ethanol in cell culture mediums pre-treated with 10 μM CCCP for 30 mins. **(A)** Western blotting analyzed mitophagy-related proteins LC3, P62, and PINK1. **(B)** Expression of Parkin protein in mitochondria and cytoplasm in Raw264.7 cells were detected by western blotting. **(C)** Expression of protein Bax, Bcl-2, and Caspase-3 were detected by western blotting. **(D)** Mitochondrial ROS was analyzed by fluorescence microplate. **(E)** The mitochondrial membrane potential (ΔΨm) was subjected to Rho123 fluorescence analysis. **(F)** Cell viability in Raw264.7 cells were measured by a CCK8 kit. Data were presented as mean ± SEM (n=5 in each group), *P < 0.05, **P < 0.01.

To further confirm whether the protective effect of MitoQ was related to mitophagy in ethanol-worsened LPS injury, the mitophagy targeted activator CCCP was used in our study. CCCP (10 μM) was added to culture medium for 30 min before ethanol exposure. First, the effects of CCCP on the mitophagic related protein was tested, as shown in **Figures 9A, B** MitoQ significantly decreased the protein level of LC3 II, PINK1, mitochondrial Parkin and increased the protein level of P62 in ethanol and LPS treated cells ethanol and LPS, which was reversed by CCCP. To clarify the effect of mitophagy on the progress of ethanol-LPS-induced mitochondrial damage, we evaluated the status of protein activation of Bax, Bcl-2, and Cleaved Caspase-3. As shown in **Figure 9C**, CCCP markedly inhibited MitoQ -induced the decrease of protein Bcl-2, and increase of protein Bax and Cleaved Caspase-3 in ethanol-LPS-treated cells. Next, Mitochondrial ROS and the mitochondrial membrane potential were analyzed. Results showed that MitoQ significantly decreased mitochondrial ROS level and improved the ΔΨm loss induced by ethanol-LPS, which were significantly reversed by CCCP, as shown in **Figures 9D, E** Subsequently, cell viability was detected by a CCK8 kit, MitoQ significantly protected against cell injury induced by ethanol–LPS, which was inhibited by CCCP (**Figure 9F**). These results suggest that mitoQ protected against ethanol and LPS-induced injury through inhibiting mitophagy in Raw264.7 cells.

## Discussion

During ALI, when critical tissue injury and exacerbation of inflammation occur, cell endogenous antioxidant system is overwhelmed by the overproduced ROS generated by various kinds of cells including macrophages, alveolar epithelial and endothelial cells, etc. and results in cell death and lung dysfunction. Previous observations indicate that activation of the inflammatory response may play a central role in the pathogenesis of most cases of ALI/ARDS (41, 42). Multiple studies indicate a correlation between the number of inflammatory cells in the alveolar spaces and resulting severity of the diseases (43–45). It is known that chronic ethanol exposure is a clinically important risk factor for the development of acute respiratory distress syndrome, the most severe form of acute lung injury (ALI) (46). However, the mechanisms by which ethanol sensitizes the lung to development of ALI are poorly understood. In the lung, the upper airway is the first checkpoint to fail in microbe clearance during ethanol-induced lung immune dysfunction. When the upper airway fails to clear inhaled pathogens, they enter the alveolar space, where they are primarily cleared by alveolar macrophages (AM) (47). With chronic ethanol ingestion, the oxidative stress pathways in the AMs are stimulated, thereby impairing AM immune capacity and pathogen clearance. Chronic ethanol could increase the susceptibility to pneumococcal pneumonia infections, which may be due to the pro-inflammatory response of AMs, leading to increased inflammation (6, 48). Ethanol activates NLRP3/caspase-1, not only in the cerebral vessel, which leads to early development of atherosclerosis (20), but also in the brain, which amplifies neuroinflammation (49). The same results were observed in our study. As shown the *in vivo* and *in vitro* models, ethanol or LPS up-regulated the protein levels of TNF-α, IL-6, and IL-1β in BALF. Furthermore, chronic ethanol treatment aggravated LPS-induced lung inflammatory factor releases as well as activated LPS-induced injury. The NLRP3 inflammasome is activated in several types of lung injury. Long-term exposure to ethanol amplifies inflammasome activation and release of IL-1β (19). In our studies, the NLRP3 inflammasome was detected by western blotting or immunofluorescence staining. We found that ethanol significantly aggravated LPS-induced activation of the NLRP3 inflammasome and release of IL-1β.

Recently, a study showed that chronic ethanol ingestion causes cells to generate large amounts of ROS (50). Binge drinking results in mitochondria oxidative damage, which induces mitochondrial dysfunction (25, 51). Meanwhile, aberrant mitochondrial ROS production is critical to NLRP3 inflammasome activation *via* oxidation of mitochondrial DNA (51). However, it is unknown whether ethanol worsened LPS-induced-activation of the NLRP3 inflammasome is related to mitochondria oxidative damage. Therefore, the mitochondria-targeted antioxidant MitoQ was added to suppress the mitochondria ROS pathways in our study. Our results showed that MitoQ effectively decreased the levels of TNF-α, IL-6, and IL-1β in BALF *in vivo* and *in vitro*. Meanwhile, MitoQ significantly inhibited activation of the NLRP3 inflammasome following the activation of caspase-1 and release of IL-1β induced by ethanol and LPS. These results suggested that MitoQ protects against lung inflammation in ethanol-LPS-induced lung injury. Subsequently, mitochondrial balance and ROS levels were detected *in vivo* and *in vitro*. Similar results were observed in our study. Ethanol promoted loss of the mitochondrial membrane potential and ROS production induced by LPS, which was significantly decreased by treatment with MitoQ. Moreover, MitoQ also decreased the ratio of Bax/Bcl-2 and protein levels of Cleaved Caspase-3. Collectively, we found that chronic ethanol sensitized the lung to injury caused by LPS by increasing damage to the mitochondria and ROS production. Meanwhile, MitoQ protected against ethanol-LPS-induced activation of NLRP3 and helps maintain mitochondrial function.

Mitophagy is a form of selective autophagy that is specific for degradation of damaged mitochondria in the lysosome. Some studies have shown that mitophagy protects against alcohol-induced liver injury and steatosis by selectively removing damaged mitochondria (25). In hepatic stellate cells from mice that received chronic ethanol treatment, alcohol aggravated liver pathological changes in the liver fiber by activating autophagy (52). However, the role of mitophagy in chronic ethanol-LPS-induced lung injury is unclear. In our study, we found that chronic ethanol consumption aggravated LPS-induced mitophagy *in vivo* and *in vitro*. Unexpectedly, mitophagy was significantly inhibited by MitoQ. To further confirm the role of mitophagy in the model, the mitophagy activation agent CCCP (53) were used in Raw264.7 cells. The data suggest that enhanced mitophagy aggravated cell mitochondrial ROS production induced by ethanol and LPS in the presence of MitoQ. These results suggest that the effect of mitophagy during the chronic ethanol expose increase in LPS-induced injury is harmful, and the protective role of mitoQ is related to the inhibition of mitophagy in Raw264.7 cells.

Not only the macrophages mentioned in this paper, but also endothelial and epithelial functions are enhanced by MitoQ when lung injury is caused by EtOH+LPS (32, 54). In two clinical trials conducted so far, MitoQ was safe and well-tolerated (55, 56). MitoQ did not harm healthy mice when given at high doses for 28 weeks. It caused no DNA damage, free radical damage, or major changes in metabolism (57) In human cells, MitoQ is 800 times stronger than idebenone (another CoQ10 analog) at preventing cell death from free radicals (58). Since MitoQ is not approved by the FDA for any condition, there is no official dose. All in all, the long-term safety profile of MitoQ is relatively unknown. Caution is warranted.

## Conclusion

In conclusion, chronic exposure to ethanol results in accumulation of ROS within the mitochondria, and activation of the NLRP3 inflammasome worsen the lung injury induced by LPS. Therefore, decreasing mitochondrial ROS is an important way to protect against EtOH-LPS-induced lung injury. Our results demonstrated that the mitochondria-targeted antioxidant MitoQ ameliorated EtOH-LPS-induced lung injury by inhibiting mitophagy and the NLRP3 inflammasome. Meanwhile, mitophagy played a harmful role in EtOH-LPS-induced lung injury. This study provides new therapeutic insights and potential treatment targets for ethanol-associated diseases.

## Data availability statement

The original contributions presented in the study are included in the article/**Supplementary Material**. Further inquiries can be directed to the corresponding authors.

## Ethics statement

The animal study was reviewed and approved by the Experimental Animal Welfare Ethics Review Committee of Wenzhou Medical University.

## Author contributions

WS, SC, and LL conducted an animal feeding experiment. LL and NW performed data analysis. WS conducted cell culture experiment. WS and SC drafted the manuscript. WS prepared figures. WS, SC, and NW participated in the manuscript preparation. XK and JY are the guarantor of this work. All authors contributed to the article and approved the submitted version.

## Funding

This work was supported by grants from the National Science Foundation of China (No. 81772450, No. 81600033), Zhejiang Provincial Medical and Health Science and Technology Project (ZY2020026) The Special Project for Significant New Drug Research and Development in the Major National Science and Technology Projects of China (2020ZX09201002).

## Conflict of interest

The authors declare that the research was conducted in the absence of any commercial or financial relationships that could be construed as a potential conflict of interest.

## Publisher’s note

All claims expressed in this article are solely those of the authors and do not necessarily represent those of their affiliated organizations, or those of the publisher, the editors and the reviewers. Any product that may be evaluated in this article, or claim that may be made by its manufacturer, is not guaranteed or endorsed by the publisher.
