# Supplementary material for: Antioxidant mitoquinone ameliorates EtOH-LPS induced lung injury by inhibiting mitophagy and NLRP3 inflammasome activation
Source: Front Immunol. 2022 Aug 18;13:973108. doi: 10.3389/fimmu.2022.973108 (PMC9436256; doi:10.3389/fimmu.2022.973108)
Supplement: Supplementary file 1 [file DataSheet_1.docx]

**SUPPLEMENTARY INFORMATION**

**Antioxidant mitoquinone ameliorates EtOH-LPS induced lung injury by inhibiting mitophagy and NLRP3 inflammasome activation**

**Wenhua Sang^1,2^** ^†^**, Sha Chen^1^** ^†^**, Lidan Lin^1^, Nan Wang^1^, Xiaoxia Kong^1*^ and Jinyan Ye^3*^**

^1^ School of Basic Medical Sciences, Institute of Hypoxia Research, Cixi Biomedical Insititute，Wenzhou Medical University, Wenzhou, China

^2^ School of Basic Medical Sciences, Zhejiang University, Hangzhou, China;

^3^ Department of Respiratory Medicine and Critical Care Medicine, The First Affiliated Hospital of Wenzhou Medical University, Wenzhou, China

*** Correspondence:**

Xiaoxia Kong

[389058669@qq.com](mailto:389058669@qq.com)

Jinyan Ye

[YJY@wzhospital.cn](mailto:YJY@wzhospital.cn)

^†^These authors have contributed equally to this work and share first authorship

**Inventory of Supplementary Information**

**Supplementary Figures**

1. Supplementary Figure A. Related to Figure 1. Expression levels of Drp1 and Mfn in HUVEC cells.

2. Supplementary Figure B. Related to Figure 1. Expression levels of Drp1 and Mfn in A549 cells.

**
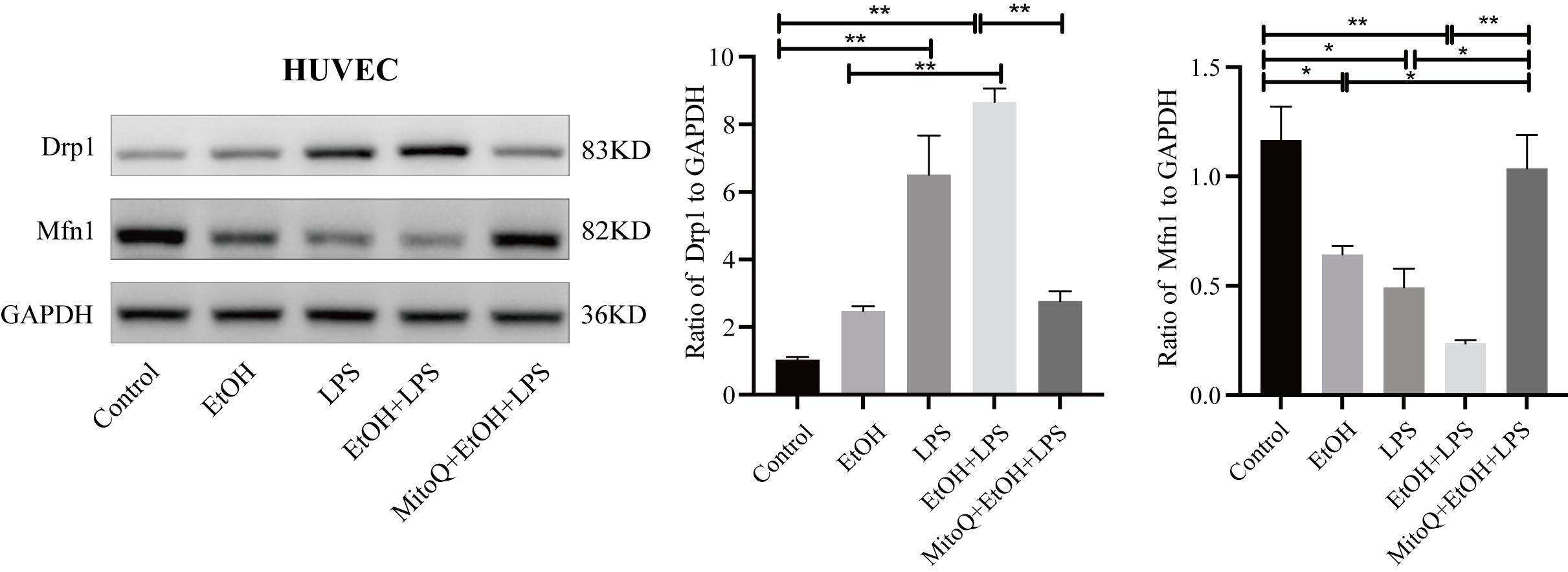
**

**Supplement Figure A:** MitoQ protects the mitochondrial function of endothelial cells. Protein expression of Drp1 and Mfn1 in the HUVEC cell was shown by western blotting. All data were presented as mean±SEM (n=5 in each group), *P < 0.05, **P < 0.01.


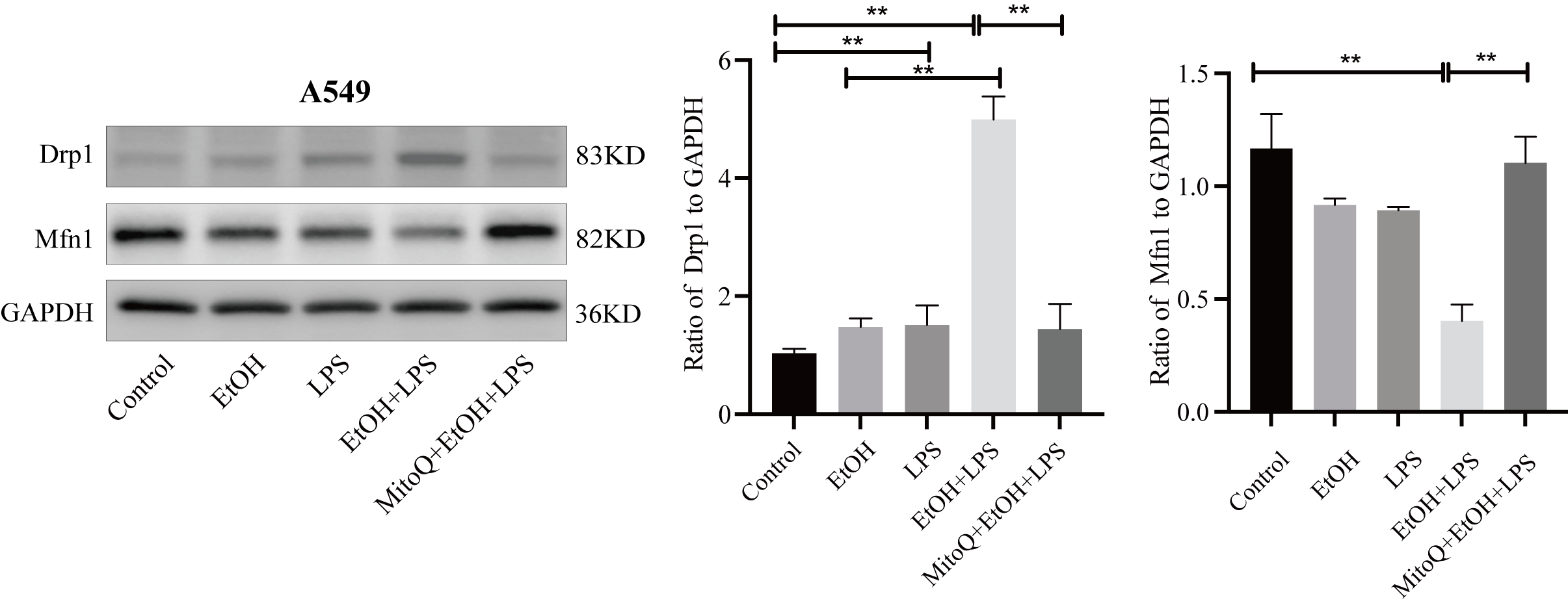


**Supplement Figure B:** MitoQ protects the mitochondrial function of epithelial cells.

Protein expression of Drp1 and Mfn1 in the A549 cell was shown by western blotting. All data were presented as mean±SEM (n=5 in each group), *P < 0.05, **P < 0.01.
